# Identifying the Needs of Primary Care Providers Caring for Breast and Colon Cancer Survivors in the Safety-Net: a Qualitative Study

**DOI:** 10.1007/s13187-022-02195-3

**Published:** 2022-07-13

**Authors:** Niharika Dixit, Gladys Rodriguez, Urmimala Sarkar, Nancy Burke, Evelin Trejo, Denise Joanna Devore, Paul Couey, Anna María Nápoles

**Affiliations:** 1 Division of Hematology/Oncology, Department of Medicine, University of California San Francisco, San Francisco, CA USA; 2 Hematology/Oncology, Stanford School of Medicine, Palo Alto, CA USA; 3 Division of General Internal Medicine, Department of Medicine, University of California San Francisco, San Francisco, CA USA; 4 Center for Vulnerable Populations, Department of Medicine, University of California San Francisco, San Francisco, CA USA; 5 School of Social Sciences, Humanities, and Arts, University of California Merced, Merced, CA USA; 6 Selby Lane, Redwood City, CA USA; 7 Division of Intramural Research, National Institute On Minority Health and Health Disparities, National Institutes of Health, Bethesda, MD USA

**Keywords:** Primary care, Cancer survivorship, Breast cancer, Colon cancer, Safety-net

## Abstract

As the number of cancer survivors continues to increase and given the shortage of oncology clinicians in safety net health care settings, primary care providers (PCPs) in these settings will increasingly provide cancer survivorship care. In order to ensure equitable care for low-income and underserved breast and colon cancer survivors, it is essential to understand the safety-net PCPs’ perspective. We conducted semi-structured, in-depth qualitative interviews with 11 PCPs working in a safety-net health care system to identify their needs in caring for cancer survivors. Interviews were audio-recorded and professionally transcribed. Two coders independently coded the interviews and conducted regular meetings until we reached consensus on the results. Analysis was based in grounded theory and performed using the constant comparative method. Thematic analysis identified six themes as follows: (1) *Cancer survivorship care can be integrated with the whole person and chronic disease care management that occurs in primary care*; (2) *PCPs’ perceptions regarding patients’ survivorship care needs and their confidence in meeting those needs*; (3) *preference for a shared care model*; (4) *coordination of care*; (5) *PCPs’ need for survivorship care education and training*; and (6) *unique issues involved in the care of older cancer survivors*. PCPs in the safety-net believe that providing comprehensive survivorship care requires coordination of care through the cancer continuum. Tools like checklists, electronic health records-based communication, and convenient electronic consultations with cancer specialists would enhance the quality of survivorship care. Respondents advocate the inclusion of survivorship care education in medical education. The continuity of care with PCPs means that they play a particularly important role in the care of older cancer survivors.

## Introduction

In 2020, there were 3.8 million breast cancer survivors and 1 million colon cancer survivors in the United States (US) [1, 2]. This number is expected to increase due to advances in cancer care and growth in the aging population. As the US population ages, the number of older cancer survivors 65 years and older is expected to increase substantially, resulting in the so-called silver tsunami [3]. Furthermore, the proportion of cancer cases that occur in ethnically diverse populations is projected to increase [4]. There is no universal health coverage in the US and health care is a mix of public and private insurance, and non-profit and for-profit health systems resulting in variable access to care. Safety-net health systems in the US provide care for low-income, uninsured or underinsured, racial and ethnic minorities, immigrants, who may have low health literacy, and limited English proficiency [5, 6]. It is hard to estimate what proportion of patients are seen in the safety-net settings; however, up to 86% of patients seen in safety-net health systems are low income, 40% are uninsured, and up to 65% are racial and ethnic minorities [7]. The projected US population shifts elevate the public health importance of efforts to ensure equitable and accessible survivorship care for all, especially underserved cancer patients seen in safety-net health systems settings [8]. Understanding the experiences and needs of primary care providers (PCPs) also called general practitioners (GPs) outside the US in taking care of cancer survivors in the safety-net setting is an important step in optimizing cancer survivorship care among vulnerable populations.

The optimal model of survivorship care has not yet been firmly established, and cancer survivors may receive care in oncology specialty clinics or primary care clinics [9]. In the US, the vast majority of cancer survivors receive care from PCPs, and cancer survivors with localized disease are more likely to receive care from PCPs [10, 11]. This includes well-described components of survivorship care: surveillance for cancer recurrence, follow-up, and management of late and long-term side effects of cancer treatments, health maintenance, communication, and coordination with other specialists [12]. PCPs with clear and concise surveillance and management guidelines achieve cancer care outcomes similar to specialty care, including cancer recurrence-related serious events and health-related quality of life [13]. Moreover, primary care practices are well suited to provide breast and colon cancer survivors with much needed education and support for lifestyle changes to improve outcomes related to cancer recurrence and other chronic diseases such as heart disease, diabetes, and respiratory diseases that may also contribute to mortality in cancer survivors [14]. Thus, the role of PCPs in survivorship care is crucial. Finally, older cancer survivors have distinct survivorship care needs as compared to younger cancer survivors and a higher need for PCPs’ involvement in survivorship care [15–17]. In safety-net health care systems that care for low-income, uninsured/underinsured cancer survivors, there are shortages of both specialists and PCPs, and resources are limited. Thus, efficient care models and coordination of care are critical in providing care for cancer survivors.

Several studies have noted that PCPs in the US feel unprepared for caring for cancer survivors and more so in a safety-net health system [9, 18]. While PCPs believe that they have the ability to care for cancer survivors, less than 40% of them feel confident about surveillance of recurrent disease [19]. Furthermore, oncologists feel that PCPs may not have the necessary skills to care for cancer survivors [9]. A survey found that PCPs have limited information about cancer survivors’ care and do not have information about details of treatments and side effects of treatment and surveillance of cancer [20]. Currently, primary care-based cancer survivorship care faces numerous challenges, including (1) poor communication between PCPs and specialists [18]; (2) oncologists’ lack of confidence in PCP-delivered care of cancer survivors [21]; (3) cancer survivors’ lack of trust in the ability of PCPs to provided cancer related care [22]; and (4) lack of medical training for PCPs on cancer survivorship care [21]. Although there is evidence of suboptimal training regarding cancer and its treatment and long-term care of cancer survivors in the primary care curriculum [23], it is not well established how and when this education should be delivered. Finally, given the distinct needs of older cancer survivors, there is a critical need for an improved understanding of the role of PCPs in the care of older cancer survivors.

There is a lack of data on the needs of PCPs in a safety-net health system who often care for racial and ethnic minority cancer survivors. Given the relative lack of attention to the delivery of cancer survivorship care in safety-net settings and the vulnerable nature of the cancer patients PCPs serve, this study was conducted as part of a mixed methods cross-sectional study to understand the attitudes and perspectives of PCPs related to caring for breast and colon cancer survivors in a safety-net health system. We conducted the quantitative component of the study to understand PCPs’ knowledge and attitudes in caring for breast and colon cancer survivors using a validated survey developed by the National Cancer Institute/American Cancer Society called the Survey of Physician Attitudes in Care of Cancer Survivors (SPARCCS) [19] which was modified for the safety-net setting. We found in our survey that about 50% of PCPs who responded (*n* = 110) agreed or somewhat agreed that PCPs have the knowledge needed to provide follow-up care for cancer survivors [2]. We also noted that except for mammograms for surveillance for breast cancer, PCPs were not familiar with the frequency of other surveillance including imaging and blood tests, and had low recognition of long-term side effects of chemotherapy. We designed the qualitative component of the study to understand in greater detail the barriers to PCP-delivered care of cancer survivors and PCP attitudes to survivorship care. Finally, we hoped to understand the needs of PCPs in order to identify interventions that could be useful in implementing PCP-delivered survivorship care in a cancer survivorship program where physician roles are well defined, thereby eliminating practice variation and providing optimal survivorship care.

## Methods

### Study Design

We designed a mixed method cross-sectional study to understand attitudes and perspectives of PCPs related to caring for breast and colon cancer survivors in a safety-net health system. The results of the quantitative survey (*n* = 110) component of the study have been published earlier [18]. We found in the quantitative survey that PCPs in our safety-net setting want to share the responsibility of care but are limited by lack of knowledge of surveillance of cancer and long-term side effects of cancer treatments. We also found that survivorship care is impacted by lack of training and lack of coordination of care between oncologists and PCPs. Informed by the survey results, in this qualitative component, we sought to understand the care of cancer survivors in a primary care setting in greater detail. We conducted semi-structured, in-depth interviews with PCPs from July 2017 to July 2018, to explore their perceptions and needs in caring for cancer survivors in a safety-net health system. Specifically, we asked their thoughts about the survivorship care needs of cancer patients in primary care, their confidence in meeting those needs, their thoughts about models for providing survivorship care, issues related to care coordination, and the care of older (aged 65 and older) cancer survivors. The Institutional Review Board at the University of California, San Francisco, approved the study. This qualitative study is reported consistent with the Consolidated Criteria for Reporting Qualitative Research [24].

### Setting and Participants

Our health network is a system of care for indigent and low-income residents in an urban setting in California. The health network includes twelve primary care clinics and a public hospital that provides primary care and specialty care. We recruited semi-structured interview participants from the PCPs who completed the initial survey [18] with a purposive sampling approach to include several different clinics. We intended to interview 15 participants or until we reached thematic saturation. We contacted potential participants by email and conducted in-person interviews at the participants’ respective primary care clinics.

### Data Collection and Analysis

We developed a semi-structured interview guide based on the initial survey questions and prior research regarding the needs of cancer survivors [18, 25]. Based on the results of the quantitative survey, we modified the interview guide specifically adding questions about communication which was noted in the survey to be suboptimal [18]. ND and GR, who are both physicians, conducted the interviews. Interviewers oriented the participants to the survivorship care of breast and colon cancer survivors and reminded them about the context of the study. The probes and additional prompts were at the interviewer’s discretion. Participants were compensated $50 in the form of gift cards for taking part in the interview. We audio-recorded the interviews which were then professionally transcribed and imported into Dedoose, a qualitative data management software. To ensure accuracy, we compared the transcripts to the audio recordings. The interviews ranged in length from 45 to 60 min.

The analysis was based in grounded theory (thematic analysis) and performed using the constant comparative method. Two researchers (ND and GR) independently read the transcribed text and identified specific codes within the interview guides using the open coding technique to create a codebook. Subsequently, the coders (ND and GR) used the codebook independently to apply the focused coding technique. Two coders discussed any discrepancies, created additional codes, and modified the codebook until consensus was reached. We ensured interrater reliability by coding the first several transcripts as a group and then individually coding and meeting together regularly to discuss data interpretation. We reached final themes through in-person meetings between the two coders, which resulted in consensus.

## Results

We contacted 15 potential participants, all of whom agreed to participate in the interviews. We conducted a total of 10 semi-structured, in-depth interviews with 11 PCPs from five different clinics. Except for one interview with two PCPs from the same practice (at the request of the participants), we conducted interviews with individual PCPs. Although a total of 15 interviews were planned, we achieved thematic saturation with 10 interviews and 11 participants; therefore, four of the PCPs who originally agreed to participate were not interviewed. Participants included 10 MDs and one nurse practitioner; two of the MDs were internal medicine residents in the primary care track. Four were family medicine providers and seven were from internal medicine. Seven participants identified as non-Hispanic white, two as Black/African American, and two as Asian American. Three of the participants were men and eight were women. Two of the physicians were in leadership positions, such as the medical director of the clinic. The characteristics of PCPs are reported in Table 1.

**Table 1:** Characteristics of primary care providers, health network, 2017–2018, *N* = 11

Characteristic		*N* (%)
Sex	Female Male	7 (64) 4 (37)
Age	20–39 40–59 60 and above	6 (55) 3 (27) 2 (18)
Primary medical specialty	Internal medicine Family medicine	7 (64) 4 (37)
Board certification	Yes No	7 (64) 3 (27)
Race	White Black/African American Asian American	7 (64) 2 (18) 2 (18)
Training	Completed In training	9 (82) 2 (18)
Provider type	MD Advanced practitioner	10 (91) 1 (9)

We identified six overarching themes relevant to the roles of PCPs in the care of breast and colon cancer survivors. We categorized the six themes as follows: (1) *Cancer survivorship care can be integrated with the whole person and chronic disease care management that occurs in primary care*; (2) *PCPs’ perceptions regarding patients’ survivorship care needs and their confidence in meeting those needs*; (3) *preference for a shared care model*; (4) *coordination of care*; (5) *PCPs’ need for survivorship care education and training*; and (6) *unique issues involved in the care of older cancer survivors*.

### Theme 1. Cancer Survivorship Care Can Be Integrated with Whole Person and Chronic Disease Care Management That Occurs in Primary Care

PCPs in our group agreed that primary care has a vital role in survivorship care for colorectal and breast cancer patients. PCPs emphasized that the whole-person care that occurs in primary care is not a separate entity from cancer survivorship care. They also noted that cancer survivorship has not received similar attention in primary care as other chronic diseases and expressed that this needs to change. For example, one PCP said, “*I would like to see cancer survivorship right up there with diabetes management. Because I think it really is – if it’s truly part of our work, but we pretend that it’s not, and we just try to wing it every time it comes up” (P3)*. One PCP also emphasized the longitudinal relationship with the patient and its importance in caring for cancer survivors.

The PCP also added, *“I don’t see cancer as a completely other entity to the other things that we’re managing in primary care. It’s another thing to be aware of, to know what the evidence shows and sort of what the guidelines are and to try a range of things” (P3)*.

Many of the PCPs compared complex disease management care that they provide, for example, management of diabetes and health maintenance as comparable to cancer survivorship care. In other words, they did not consider survivorship care separate from the care they already provided for chronic medical conditions. Checklists are frequently used by PCPs to manage chronic diseases such as diabetes. They suggested that a similar approach can be utilized to provide standardized, high-quality cancer survivorship care as used in chronic disease management in primary care settings. PCPs acknowledged the lack of formal workflows around cancer survivorship care emphasizing the need to create such resources and a checklist. For example, one PCP stated, “*I have a specific checklist that I – for people with diabetes and people with cirrhosis, for example. And it’s the same thing with this if you think about it. Someone is in remission from breast cancer; these are the things that you need to do.” (P1)*.

### Theme 2. PCPs’ Perceptions Regarding Patients’ Survivorship Care Needs and Their Confidence in Meeting Those Needs

Regarding the needs of cancer survivors, PCPs felt that cancer survivors need information about their diagnosis, treatment, and surveillance. One PCP stated, *“I think it’s great for them to know their surveillance recommendations. It’s no different than other medical conditions like HIV and diabetes. The more a patient can be empowered to understand their conditions or their recommendations, the better in my mind” (P3)*. Furthermore, PCPs also thought that cancer survivors should receive health maintenance information, such as physical activity and diet.

PCPs were aware of the delayed and long-term adverse effects of cancer treatments and the need to manage symptoms but did not always feel comfortable providing this care. They expressed a lack of knowledge of the natural history of long-term side effects such as neuropathy and the inability to counsel patients regarding these side effects. For example, one PCP said, “*I have a patient with neuropathy. That seems more common now with some cancer treatments. And so, again, is this going to get better? Is it not? What should you expect?” (P3)*.

PCPs recognized the impact on the psychosocial health of cancer survivors and the need for support from behavioral health and support groups. One PCP stated, *“I would say depression might be high. I would definitely screen them. And then they probably could benefit from therapy – like psychotherapy – just to be able to talk about their experience, ‘cause it can be an isolating experience. A support group would be good.” (P7)*. PCPs also noted their familiarity with behavioral health interventions and the embedded behavioral health team in primary care clinics in their network which made them comfortable with managing the psychosocial impact of cancer survivors’ care. To illustrate, one PCP said, *“The behavioral health team here at the Health Center does provide a lot of support for our patients that I don’t necessarily feel overwhelmed by it” (P3)*.

Concerning their confidence in meeting the cancer surveillance needs of cancer survivors, PCPs expressed confidence in their ability to diagnose and order an initial workup for recurrent disease; however, this was accompanied by some anxiety that they may miss some recurrences. They felt reassured by their ability to reach out and consult oncology as needed, highlighting the need for collaborative care approaches. For example, one PCP stated, “*I think I feel good about initial diagnostic imaging. And I think if I have a suspicion, I will often send a referral to oncology just to make sure I’m not missing anything” (P6)*.

### Theme 3. Preference for a Shared Care Model

PCPs were reluctant to assume complete care for cancer survivors immediately and expressed support for the shared care model where the PCPs and oncologists collaboratively care for cancer survivors for a period of time. PCPs assume full care for some early-stage colon cancer survivors in our health care setting who only undergo surgery and do not receive chemotherapy similar to a risk stratified model of survivorship care. For most other cancer survivors, they preferred a shared care approach. For example, one participant said, *“I like the idea of it being both the oncologist and the primary care doctor for a certain amount of time after someone is deemed in remission. But then the further out someone is, the less likely they are to have a recurrence. And at some point, I think it’s – it should go back to the primary care doctor” (P3)*.

PCPs saw their role in the shared care model as reinforcing some of the messages from oncology as fundamental to a team-based approach. One participant said, *“And I think the reason why I feel so strongly that primary care is important is because we take care of patients longitudinally, and an oncologist is only gonna manage the cancer and diseases related to that cancer and the side-effects and stuff. But I would – will take care of that patient’s cancer and everything else that’s going on in that patient’s life, which is why I think it’s so important to also involve primary care” (P1)*.

### Theme 4. Coordination of Care

PCPs strongly emphasized the need for coordination of care. They appreciated receiving a clear note from oncology specialists outlining the plan of care including surveillance imaging. PCPs felt more comfortable assuming survivorship care if they had access to a clear plan of care from oncology. To illustrate, one participant stated, *“I recently saw a patient who was discharged from his oncologist. And that note said, ‘Patient needs X, Y, or Z.’ I can’t remember the exact, but maybe a CT scan every year. ‘And then send him back for X, Y, and Z symptoms.’ So that seemed pretty clear to me, and I felt comfortable taking over for that patient” (P7)*.

Participants also felt that as PCPs, they should be aware of and have access to the information given to their patients by oncology or other specialties such as survivorship care plans and treatment history. PCPs expressed that communication is suboptimal during the active cancer treatments. For example, one PCP stated about active treatment for cancer, *“I make a referral, all these studies happen, and then patients get started on treatment. And there’s really not a lot of dialogue between the specialist and the PCP” (P2)*. They expressed that consistent communication during the active treatment is also likely to make them feel more familiar with cancer care. A consistent email or electronic alert during treatment would make them more comfortable providing care for cancer survivors after treatment. PCPs expressed that standardized note templates may also be a straightforward communication method. For example, one PCP stated, “*I think that if there was a clear note template, right, that had all the information, and then sensible patient materials, referral to the care program*” (P8).

PCPs expressed a desire for as-needed access to oncologists and specialist care in order to feel comfortable in providing care to cancer survivors. PCPs endorsed an existing electronic consultation system (e-referral) that promptly addressed their questions by subspecialists as an essential resource. For example, one PCP said, “*E-referral is a big help. Just being able to ask the question. That’s where I get most of my help” (P9)*.

### Theme 5. PCPs’ Need for Survivorship Care Education and Training

Most PCPs agreed that they require additional training and education in survivorship care. PCPs suggested models such as continued medical education (CME) and web-based education and supported incorporating cancer survivorship care training in medical school and residency. For example, one PCP said, “*I think it should be provided during training. I think it’s a big gap that it’s not” (P2)*.

PCPs stated that cancer-related medical education focuses on treatment to the exclusion of survivorship care. To illustrate, one PCP noted, *“I don’t think it’s something that we’re really taught ever, formally. When do we learn that? In medical school, we learn about cancer. We learn about the treatments, but we never talk about what happens afterward” (P9)*. PCPs’ perspectives included providing survivorship care earlier in medical education and then reinforcing it during practice. For example, a PCP mentioned, *“First bring it into the training and then incorporate it into just family practice, internal medicine, and primary care practice on CME” (P3)*. Most PCPs were not familiar with survivorship care educational resources for the medical professionals. Although some of them had heard of the American Cancer Society, they were not aware of local support groups or other organizations that play an essential role in the care of cancer survivors.

### Theme 6. Unique Issues in Caring for Older Cancer Survivors

We asked PCPs about their perspectives on caring for older cancer survivors since most cancer survivors have a longitudinal relationship with their PCPs. PCPs asserted that they take in to account pre-existing comorbidities and the long-term effects of cancer treatments when managing other non-cancer chronic conditions. Similarly, the surveillance for cancer is impacted by other comorbidities. To illustrate, one PCP said, “*We become a little bit more lenient, in terms of control of chronic diseases and things like that. So I think it, you know, it really depends on also how well the patient recuperates from surviving cancer. Because some I think do really well. And, you know, it feels okay to be as proactive and aggressive as before, in terms of controlling their chronic care diseases. And then, others I think decompensate so much that you kind of pivot in a different way.” (P2)*.

PCPs stated that their long-term relationships with older cancer survivors facilitate palliative care discussions. When discussing palliative care, they consider the patient’s cancer history, goals of the cancer treatment, risk of cancer recurrence, and other comorbid conditions in addition to age. For example, one PCP said, *“But it depends on precisely how old they are and what their other comorbidities are. Because many times you may be having a palliative care discussion” (P3)*.

### Recommendations for Integrating PCPs in Survivorship Care

Synthesizing across different identified themes, the following specific recommendations emerged to integrate PCPs in survivorship care: (1) checklists for cancer survivorship care similar to checklists used in other chronic conditions such as diabetes to provide survivorship care; (2) leveraging electronic health record tools including structured notes and electronic consultations to facilitate information sharing between PCPs and specialists on surveillance, long-term and delayed side effects, psychosocial effects, and health maintenance; (3) structured transition of care with communication at different time points during cancer continuum; (4) introduction of survivorship care education earlier in training for PCPs, concurrent with education on diagnosis and treatment, followed by ongoing education including CME lectures and short question CME in journals targeted to PCPs; (5) guidelines co-created or endorsed by organization that are more familiar to PCPs; (6) tailored survivorship care for older adults based on cancer characteristics, comorbidities, and overall health status leveraging longitudinal relationships between PCPs and cancer survivors.

## Discussion

Through qualitative interviews in this study, we explored the perspectives of PCPs in a safety-net health network about the survivorship care needs of colorectal and breast cancer survivors, their confidence in providing such care, their thoughts about care coordination with oncology specialists, preferred models of care, and unique issues with respect to providing cancer survivorship care for older adults. Patients, according to PCPs, require information about their diagnosis, treatments, and surveillance requirements, as well as assistance with maintaining healthy lifestyles and managing any psychosocial distress caused by cancer. In general, PCPs felt that improved medical education and training on cancer survivorship care is needed. They perceived that better care coordination between oncologists and PCPs through structured systems approaches (e.g., structured notes and survivorship care plans, e-consults) would facilitate better survivorship care. PCPs’ beliefs about the need for better care coordination aligned well with their preference for a shared care model that acknowledges the important roles of PCPs in managing the long-term and complex care of cancer survivors who may have significant comorbid chronic conditions, especially if older in age.

Similar to prior studies [9, 19], PCPs in this study identified their role as crucial in cancer survivorship care and expressed a preference for providing this care. They were familiar with cancer survivorship care and the needs of cancer survivors, including cancer surveillance, managing adverse effects of treatment, psychosocial needs, and information needs of cancer survivors. PCPs in our study emphasized that their longitudinal relationships with the patients allowed them to focus on health maintenance and preventive care, essential components of cancer survivorship care.

PCPs also expressed a preference for a shared care model for a limited time. They expressed that this shared care model [26], where cancer survivors are co-managed with oncology specialists for a limited amount of time, should be utilized for patient and provider education, communication, and ongoing care coordination. This transition period should ensure that survivors are not “lost in transition” from oncology to primary care. PCPs felt comfortable caring for cancer survivors if there was a clear plan from the oncologist and straightforward access to an oncologist for consultation as needed, highlighting a need for clear communication and ongoing communication and coordination. While survivorship care plans are resource and time intensive [27] and have limited evidence in terms of improving outcomes [28], checklists for cancer survivorship similar to diabetes and cirrhosis management could be more practical and acceptable. Electronic medical records can easily facilitate this coordination of care with the shared plan of care, utilizing checklists and an electronic consultation and referral system.

During the active cancer treatment phase, PCPs also reported feeling excluded from their patients’ care. This gap emphasizes the critical need for PCPs to receive ongoing diagnostic and treatment information during the active cancer treatment phase. Consistent information flow without overwhelming PCPs is needed so that PCPs feel better prepared and more comfortable taking care of the patient once the active phase of treatment has been completed [29].

Finally, PCPs identified cancer survivorship education and training as essential factors in the care of cancer survivors. Based on our results, this education should begin at the medical school and residency level and then be available on demand with ongoing educational courses like continuing medical education lectures/webinars (CME) or short CME vignette style questions through journals targeted to PCPs. This is similar to Nekhlyudov et al. [29] who proposed that early integration of survivorship care education helps build an education framework on which subsequent information about cancer survivorship can be added and is critical to the integration of survivorship care in primary care practice.

Our study highlights the crucial expertise of PCPs in the care of chronic disease management, multimorbidity, and a focus on health maintenance that can be leveraged in the long-term care of cancer survivors. PCPs have taken over care for diseases once considered the domain of specialists, such as HIV, hepatitis C, and diabetes mellitus and are well positioned to care for cancer survivors. This has been supported by interventions like teleconsultations where electronic consultations between PCPs and HIV specialists allow PCPs to provide HIV care independently and reduce formal referrals [30]. The project Extension for Community Healthcare Outcomes (ECHO) demonstrates the success of an ongoing tele-mentoring model which allowing PCPs to provide high-quality specialty care including hepatitis C, HIV, diabetes and cancer [31–34]. Additional resources that can be helpful to PCPs include the dissemination of professional guidelines for cancer survivorship care. Finally, most survivorship care guidelines are established by cancer care organizations; many PCPs in safety-net systems are not aware of National Comprehensive Cancer Network (NCCN) and American Cancer Society guidelines for cancer survivorship care. It is important that these guidelines are developed collaboratively and endorsed by organizations that PCPs are likely to be more familiar with, such as the American Academy of Family Physicians (AAFP) and the American College of Physicians (ACP).

As safety-net health care organizations struggle with a shortage of PCPs and specialists [35], one of the solutions proposed by participants is similar to risk-based surveillance [36, 37] which takes into account the stage of cancer, treatment received, the estimated risk of recurrence, and patient preferences to determine the optimal cancer survivorship care follow-up. Risk-based cancer survivorship care has been implemented in the UK based on cancer survivors’ needs [37]. For example, cancer survivors with early-stage cancer treated with surgery alone can be cared for by PCPs using a cancer care surveillance checklist. Intermediate-risk cancer survivors with moderate risk of recurrence and after a period of shared care are transitioned to PCPs. Furthermore, high-risk cancer survivors who are considered to be high risk of recurrence and have significant oncologic care needs may benefit from indefinite co-management with oncologists and PCPs [37].

Our study has several limitations. Importantly, our study was limited to one safety-net health network and may not generalize to other safety-net settings. However, based on the literature, the challenges reported by our PCPs are not unique to our health network. Similarly, the solutions proposed by the PCPs in this study may be applicable to other health care settings and future studies can establish their broader relevance. Furthermore, although we reached thematic saturation after interviewing 11 participants, our results may not reflect a complete or comprehensive view of PCPs’ needs or perspectives, and important themes could have been missed. Finally, our study was conducted in the US and may not be generalizable to other health care systems worldwide.

In conclusion, through qualitative semi-structured interviews, we elicited rich data on the perspectives and needs experienced by PCPs in taking care of cancer survivors. These findings indicate that caring for cancer survivors in the safety-net setting requires improved coordination between oncology and primary care providers and greater efforts to train the primary health care workforce for optimal care of cancer survivors. Our findings can be used to inform interventions aimed at improving cancer survivorship care delivery in primary care settings, including those provided by safety-net providers.

## Data Availability

Available with authors.

## Declarations

### Disclaimer

The contents and views in this manuscript are those of the authors and should not be construed to represent the views of the National Institutes of Health.
